# Editorial: Individual and cultural differences in sustainable consumer behavior

**DOI:** 10.3389/fpsyg.2023.1147626

**Published:** 2023-02-21

**Authors:** Ahmad Daryanto, Zening Song, Didier Soopramanien

**Affiliations:** ^1^Lancaster University Management School, Lancaster University, Lancaster, United Kingdom; ^2^International Business School, Beijing Foreign Studies University, Beijing, China; ^3^International Business School Suzhou, Xi'an Jiaotong Liverpool University, Suzhou, China

**Keywords:** pro-environmental behavior, sustainable behavior change, individual characteristics, consumer behavior, consumer context

This editorial reflects upon and presents the contributions of the papers that have been chosen to appear in this Research Topic on the theme of “*Individual and cultural differences in sustainable consumer behavior*.” Our endeavor with this Research Topic is to address the environmental problems associated with consumer behaviors, practices and responses to policy initiatives and strategic actions taken by governments and firms, respectively. To address such pressing issues, we regard that this Research Topic particularly showcases how individual differences, from a consumer behavioral perspective, unpacks and illustrates the complexity that underpins changes in behaviors and practices toward a sustainable lifestyle.

In that context, the paper by Dreijerink et al. is concerned with lifestyle changes and, of particular interest, they focus on the level of effort associated with different forms of pro-environmental behaviors. Personal motivation has been studied before but its role in shaping how certain groups perceive the level of effort has not been given enough attention prior to their work. As perceptions shape our actions, there are some valuable policy lessons that emerge from their work how to engage consumers in more sustainable behaviors. The relationship that is uncovered in their research implies that we should start by encouraging individuals to engage in activities that fit with their motivation level and then engage them progressively in higher effort pro-environment behaviors.

The promotion of cultural heritage in tourism can play an important role in sustainable development in particular by promoting and encouraging visitors to visit and develop an affinity with sites that expose the wonders of nature, its fragility and thus the need to protect such places. The paper by Zhou et al. study the role of authenticity and specifically authenticity in heritage tourism in the context of a heritage site in China, namely Dujiangyan Irrigation System. They find that authenticity directly and indirectly affects tourists' revisit intention through memorable tourism experience and attachment to the place.

Knowledge that consumers have and how they use and interpret new information is crucial when the objective is to change current consumption behaviors. Three papers in this Research Topic fall under this remit but, importantly, address the focal issue concerning how differences in individual characteristics, attitudes and motivations interplay with consumers' interpretation of information. Not all consumers are willing or know how to search for information when it comes to changing behavior. In such circumstances, they often rely on cues to reduce the cost of searching. The paper by Wang et al. start with the contention that consumers might use retailer reputation as a cue in deciding whether to choose products that are branded as green. The authors furthermore propose that these effects will vary across different types of products, specifically whether green products are low or high involvement. In relation to the theme of the Research Topic on individual differences in consumer behavior, the effect of retailer reputation on whether consumers intend to buy such products is mediated, respectively, by consumer attitudes toward the environment and the regulatory focus characteristics of consumers.

Access based consumption, such as car sharing, is seen as one of the ways in which consumers can benefit from goods and services sustainably. However, because consumers do not own such products, they might care less about them whilst using them. Other consumers may complain about these products not being in good condition and consequently may not use them again and firms have to bear additional costs related to repairs and cleaning. Fu and Xu study the effectiveness of different types of messages (i.e., framing of messages effect) sent by car sharing companies in deterring intention to misbehave whilst using their services. Interestingly, whilst consumers do not own these cars, the authors consider that a sense of ownership, i.e., psychological ownership, can moderate the effect of these different types of messages. Consumers that develop a sense of ownership for products-that they do not even own-react differently to different message appeals (e.g., rational or emotional) compared to those who have not developed such a sense of ownership which then affects who will and will not misbehave whilst using products which others will also use later.

The third paper within that remit of information use and interpretation is by Xiao et al.. The starting premise of the study is that the amount of information and the way in which the information is designed on the packaging of products can influence how consumers perceive whether a product is environmentally friendly or not. This is underpinned by what is known as visual density of information and how consumers use and interpret the information presented to them. The visual density of information may be seen by consumers to be linked to the cost of producing the item too. Consumers may consequently think that such products are not as green as they are meant and intended to be: perceived greenness. Interestingly, and of relevance to the theme of the Research Topic, the authors consider that not all consumers will make such a connection between visual density and perceived cost of production. Different thinking styles, and in particular whether consumers are more or less likely to be holistic thinkers, moderate the aforementioned relationship. The thinking style of consumers influences how they initially interpret the visual density of information.

This Research Topic illustrates that research on individual and cultural differences in behavior remains important in our endeavor to better understand and achieve environmentally friendly behavioral changes. As mentioned earlier, Governments and firms are enacting different policies and actions that are intended to promote and motivate consumers to engage in pro-environment behaviors. The range of papers illustrates that the effectiveness of these policies and actions are highly dependent on the level of and the type of heterogeneity in consumer characteristics, attitudes, and behaviors. Thus, we contend to end that there remain many other avenues for further research in particular those that will focus on further unpacking how the realities of consumption interact with and reveal individual differences in consumption behavior.

## Author contributions

All authors listed have made a substantial, direct, and intellectual contribution to the work and approved it for publication.

